# Tidal breathing parameters measured by structured light plethysmography in children aged 2–12 years recovering from acute asthma/wheeze compared with healthy children

**DOI:** 10.14814/phy2.13752

**Published:** 2018-06-21

**Authors:** Hamzah Hmeidi, Shayan Motamedi‐Fakhr, Edward K. Chadwick, Francis J. Gilchrist, Warren Lenney, Richard Iles, Rachel C. Wilson, John Alexander

**Affiliations:** ^1^ Institute for Science and Technology in Medicine Keele University Stoke‐on‐Trent UK; ^2^ PneumaCare Ltd. Ely Cambridgeshire UK; ^3^ University Hospitals of North Midlands Stoke‐on‐Trent UK; ^4^ Addenbrooke's Hospital Cambridge UK

**Keywords:** Acute asthma, bronchodilator, children, structured light plethysmography

## Abstract

Measurement of lung function can be difficult in young children. Structured light plethysmography (SLP) is a novel, noncontact method of measuring tidal breathing that monitors displacement of the thoraco–abdominal wall. SLP was used to compare breathing in children recovering from an acute exacerbation of asthma/wheeze and an age‐matched cohort of controls. Children aged 2–12 years with acute asthma/wheeze (*n* = 39) underwent two 5‐min SLP assessments, one before bronchodilator treatment and one after. SLP was performed once in controls (*n* = 54). Nonparametric comparisons of patients to healthy children and of pre‐bronchodilator to post‐bronchodilator were made for all children, and also stratified by age group (2–5 vs. 6–12 years old). In the asthma/wheeze group, IE50_SLP_ (inspiratory to expiratory flow ratio) was higher (median 1.47 vs. 1.31; *P *= 0.002), thoraco–abdominal asynchrony (TAA) and left–right asynchrony were greater (both *P* < 0.001), and respiratory rate was faster (*P* < 0.001) than in controls. All other timing indices were shorter and displayed reduced variability (all *P* < 0.001). Variability in time to peak inspiratory flow was also reduced (*P* < 0.001). Younger children showed a greater effect than older children for TAA (interaction *P* < 0.05). After bronchodilator treatment, the overall cohort showed a reduction in within‐subject variability in time to peak expiratory flow only (*P* < 0.001). Younger children exhibited a reduction in relative contribution of the thorax, TAA, and variability in TAA (interaction *P* < 0.05). SLP can be successfully performed in young children. The potential of SLP to monitor diseases such as asthma in children is worthy of further investigation. ClinicalTrials.gov identifier: NCT02543333.

## Introduction

Effective management of asthma and other respiratory conditions relies on accurate assessment of lung function (Beydon et al. 2007; van den Wijngaart et al. 2015). Although spirometry is the gold standard (Global Initiative for Chronic Obstructive Lung Disease, 2010), it is often not suitable for young children who may be unable or unwilling to perform forced breathing maneuvers (van den Wijngaart et al. 2015). An alternative strategy could be to measure breathing patterns at rest (i.e., “tidal breathing”). Existing methods for assessing tidal breathing include pneumotachography (PNT) and respiratory inductive plethysmography (RIP). Both techniques can be used in young children (Stick et al. 1992; Bates et al. 2000), but practical drawbacks have limited their use clinically. Specifically, the use of a mouthpiece or mask in PNT may cause individuals to alter their normal breathing pattern, while slippage of the transducer bands used in RIP can affect the accuracy of data (Weissman et al. 1984; Stick et al. 1992; Caretti et al. 1994; Laveneziana et al. 2015a). Furthermore, although some studies have reported respiratory disease‐related changes in certain tidal breathing parameters, there is no agreement on which parameter(s) should be routinely assessed (Kuratomi et al. 1985; Brack et al. 2002; Schmalisch et al. 2005).

Structured light plethysmography (SLP) is a novel light‐based technique enabling detailed assessment of tidal breathing patterns over consecutive breaths. Unlike PNT and RIP, SLP is a noncontact method that does not require the use of a mouthpiece, nose clip, or other device. Instead, SLP measures movement (or “displacement”) of the anterior thoraco–abdominal (TA) wall. SLP is performed while the child is breathing normally and therefore can be performed in infants and very young children. Timing indices of tidal breathing such as respiratory rate (RR), inspiratory time (tI), and expiratory time (tE) measured by SLP have shown good agreement with PNT (Motamedi‐Fakhr et al. 2017a).

In this observational cohort study, we compared SLP‐measured tidal breathing parameters in children recovering from an acute exacerbation of asthma or wheeze and receiving bronchodilator medication with an age‐matched group of healthy controls. We also compared these effects in younger children (aged 2–5 years), who are generally considered to be too young to perform spirometry, with those in older children (aged 6–12 years).

## Materials and Methods

### Participants

The study recruited children aged 2–12 years admitted to hospital wards following an acute exacerbation of asthma (or breathing difficulties/wheeze in those without a formal asthma diagnosis) between March 2014 and June 2015. As part of standard care, these children received regular inhaled bronchodilators with the frequency of treatment dependent on the severity of their condition. Children were recruited between days 1 and 3 after admission when they were in the recovery phase of an acute attack, on a treatment frequency of 3‐h or longer regular salbutamol MDI, and, in their clinician's opinion, were well enough to participate. Results from the asthma cohort were compared with those from a group of healthy children aged 2–12 years without a diagnosis or symptoms of asthma or other respiratory condition.

Children were excluded from the study if they had significant comorbidity or chest wall abnormality, obstructive sleep apnea, a body mass index >40 kg/m^2^, any acute or chronic condition that restricted his/her ability to participate, or they were unable to comply with the protocol. The study was performed at the Royal Stoke University Hospital (Stoke‐on‐Trent, UK) and Addenbrooke's Hospital (Cambridge, UK).

### Study design

Once well enough, children recovering from an acute exacerbation of asthma/wheeze underwent two 5‐min SLP assessments. The first took place 5–10 min before administration of a bronchodilator, which was given as part of standard treatment and at a time determined by their clinician as necessary for their care. The number of bronchodilator treatments administered prior to this varied between children according to clinical need. The second SLP assessment occurred approximately 10–15 min after bronchodilator administration. SLP was performed once in the healthy children. A research nurse provided distraction during the procedure by means of a cartoon video viewed on a tablet so that subjects remained as still as possible.

The study (ClinicalTrials.gov identifier: NCT02543333) was conducted in line with the International Conference on Harmonisation Good Clinical Practice guidelines and was approved by the UK Health Research Authority National Research Ethics Service (reference number 11/EE/00/37). Parents/guardians provided written informed consent.

### SLP procedure and data analysis

**Figure 1:**
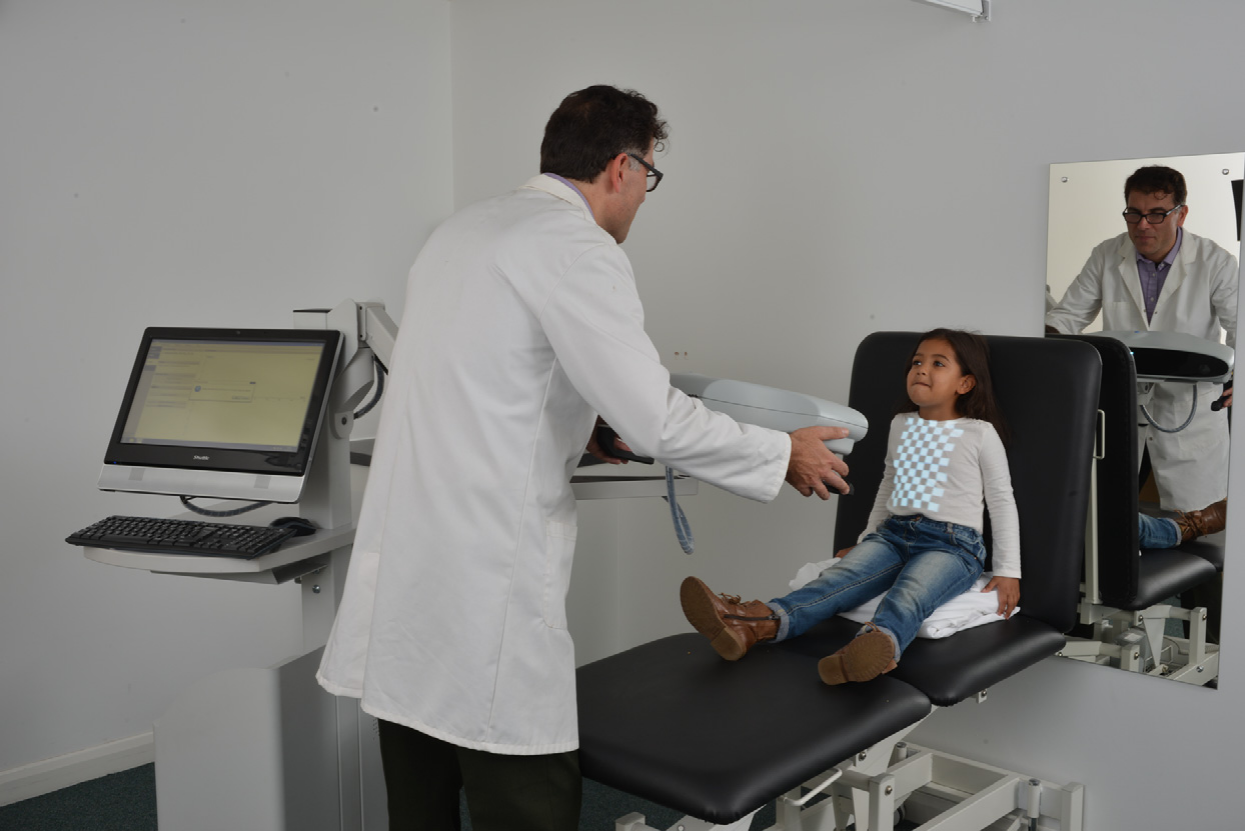
Principles of structured light plethysmography. A grid of light is projected onto the thoraco–abdominal (TA) wall of a participant. The changes in the grid pattern that occur during breathing are recorded by two cameras, which are located in the scanning head. These changes are translated into a virtual surface that corresponds to the shape of the subject's TA wall. Tidal breathing timing indices are then calculated using the one‐dimensional movement over time trace generated from the average axial displacement of the grid. The subject in the photo was a volunteer and not a study participant.

Tidal breathing was measured using an SLP device (Thora‐3Di™, PneumaCare Ltd., Ely, Cambridgeshire, UK), as previously described (Hmeidi et al. 2017). Briefly, a grid of light was projected onto the TA wall and changes in the grid pattern were recorded using two digital cameras in the scanner head of the SLP device (Fig. 1). Images captured by the digital cameras were translated into a virtual surface representing each child's TA wall. To ensure data were as artifact‐free as possible, it was decided prior to analysis that a dataset would be excluded if data artifacts affected more than 50% of captured respiratory cycles. Individual breaths were detected using a breath detection algorithm based on the works of Bates et al. (2000) and Schmidt et al. (1998) (MATLAB^®^, R2015b; Mathworks, Natick, MA, USA).

### Tidal breathing parameters

Methods for calculation of tidal breathing parameters obtained from SLP have been reported in detail elsewhere (Hmeidi et al. 2017). The categories of parameters are described briefly below.

#### Timing indices

Timing indices and ratios (RR, tI, tE, total breath time [tTot], and tI/tE and tI/tTot) were obtained from the average TA wall displacement versus time signal, which is a measure analogous to volume.

#### Flow‐based parameters

Other parameters were derived from the rate of TA wall displacement (i.e., the first derivative of the displacement signal). These SLP parameters are similar to certain conventional tidal breathing parameters as TA wall displacement rate is analogous to flow. Therefore, similar nomenclature is used to describe SLP parameters derived from displacement rate, with the added suffix “_SLP_”. These parameters include time to reach peak tidal expiratory flow over tE (tPTEF_SLP_/tE), time to reach peak tidal inspiratory flow over tI (tPTIF_SLP_/tI), and IE50_SLP_. The latter parameter was calculated by dividing SLP‐derived tidal inspiratory flow at 50% of inspiratory volume (TIF50_SLP_) by tidal expiratory flow at 50% of expiratory volume (TEF50_SLP_).

#### Regional parameters

Further SLP parameters were derived from regional displacements of the TA wall and calculated by dividing the 3D reconstruction of the TA wall into two equally sized sections. Division lines for the separation of regions were a horizontal line at the xiphisternum (for upper and lower comparisons) and a vertical line at the sternum (for right to left comparisons). Relative contribution of the upper region (thorax) to each breath (rCT) was expressed as a percentage of the total TA movement. TA asynchrony (TAA) was expressed as the phase difference in degrees between the upper and lower regions. Left–right hemi‐thoracic asynchrony (HTA) was expressed as the phase difference in degrees between the left and right hemi‐thoracic regions.

### Statistical analysis

These data are the first reported using SLP in young children. Therefore, it was not possible to perform a priori power calculations.

SLP measures multiple sequential breaths per assessment. For each assessment, the median and interquartile range (IQR) of each tidal breathing parameter were calculated. These values are presented in the results with the prefix “m” to denote median and “v” to denote IQR (i.e., the within‐subject variability). Individual data for each parameter and its variability were then combined for each cohort and summarized by their median and IQR.

A Mann–Whitney *U* test was used to compare each “m” and “v” parameter in healthy children and those with acute asthma/wheeze (both before and after bronchodilator administration). A robust two‐way ANOVA (raov in the R package Rfit) was used to test for significant interactions between these effects and age (Kloke and McKean 2012). A paired Wilcoxon signed‐rank (WSR) test was used to assess the effect of bronchodilator in children with asthma/wheeze. A Mann–Whitney *U* test of the differences (post – pre‐bronchodilator) was used to compare these effects in younger and older children. The ability of SLP parameters to differentiate children with asthma from those without, and also to detect a response to bronchodilator, was further assessed by calculating the common language effect size (CLES) for all parameters that demonstrated a significant difference between groups. As this was an exploratory study, no adjustments were made to *P*‐values for the multiple tests conducted.

## Results

**Figure 2:**
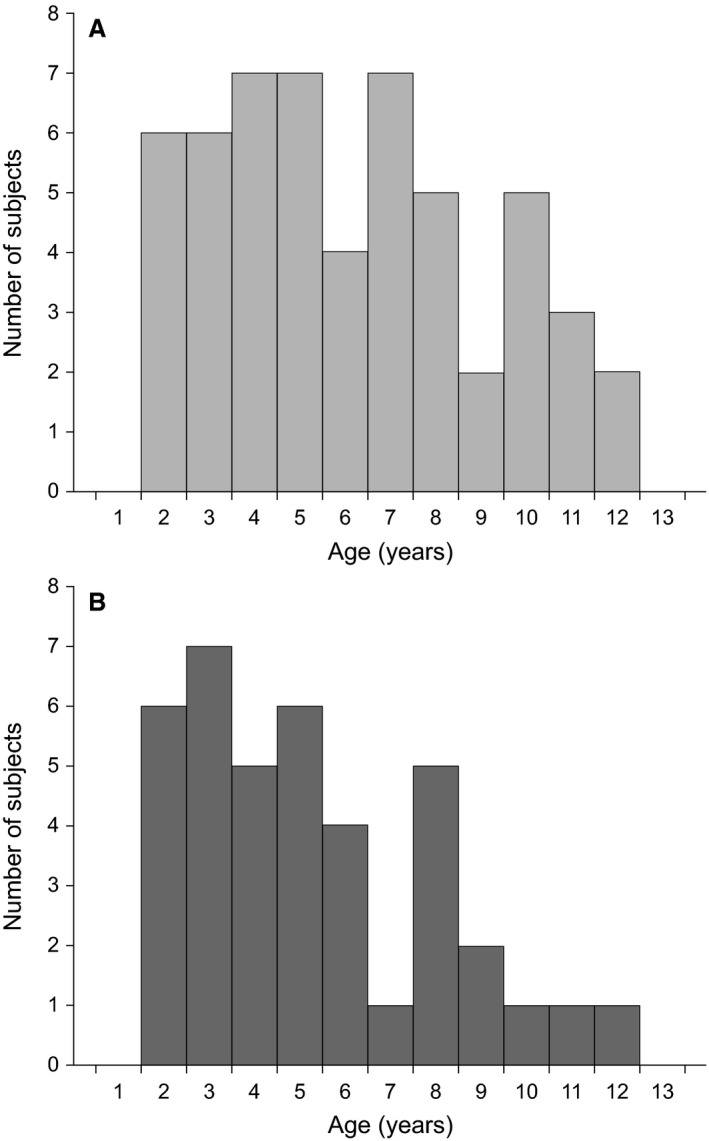
Age distribution of the participants in the (A) healthy and (B) acute asthma/wheeze groups.

Thirty‐nine children with acute asthma/wheeze (26 with a formal diagnosis of asthma) plus 54 age‐matched healthy controls were eligible for this study and provided evaluable data for analysis using the strict criteria outlined above. The age distribution and demographics of the two cohorts included in the analysis were similar (Fig. 2; Table 1). The success rate for the SLP procedure (defined as the number of measurements providing evaluable data divided by the total number of measurements performed) was 87.8% (137/156). When assessed according to age, the success rate in older participants (aged 6–12 years inclusive) was 93.7% (59/63) and in the younger preschool participants (aged 2–5 years inclusive) was 83.9% (78/93). The reason for exclusion of data was poor quality in one or both (in the case of the acute asthma group) of the datasets, caused by interference from movement, light, or creases in the t‐shirt.

**Table 1:** Participant demographics

	Healthy children (*N* = 54)	Children with acute asthma/wheeze (*N* = 39)
Gender (male: female), *n*	33:21	26:13
Age, years	6.1 (2.9)	5.2 (2.7)
Age groups (2–5: 6–12 years), *n*	26:28	24:15
Height, cm	116.5 (21.0)	114.1 (18.3)

Data are mean (standard deviation) unless otherwise indicated.

**Figure 3:**
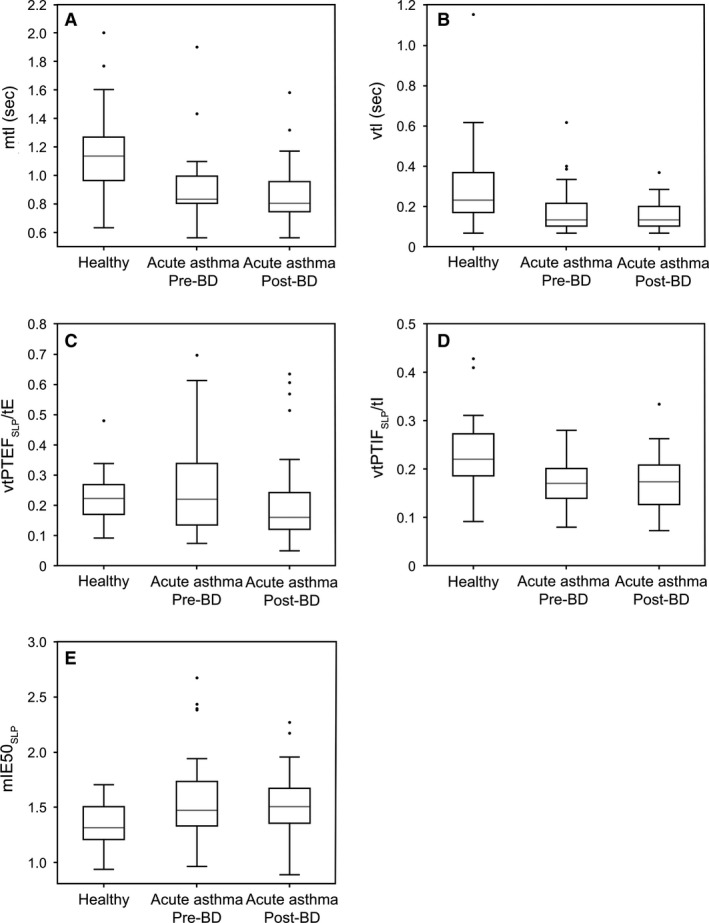
Two of the nine timing‐based parameters (mtI [A], vtI [B]), and three flow‐based parameters (vtPTEF_SLP_/tE [C], vtPTIF_SLP_/tI [D], and mIE50_SLP_ [E]) differed between healthy children (*n* = 54) and those with asthma/wheeze (*n* = 39) both pre‐ and post‐bronchodilator administration. The reduction in vtPTEF_SLP_/tE in the children with asthma following bronchodilator administration is also illustrated (C). The gray line indicates the median value, the rectangle spans the interquartile range, and the black whiskers indicate the minimum and maximum values (excluding the outliers indicated by the black circles). BD, bronchodilator; IE50_SLP_,SLP‐derived tidal inspiratory flow at 50% of inspiratory volume divided by tidal expiratory flow at 50% of expiratory volume; m, median; SLP, structured light plethysmography; tE, expiratory time; tI, inspiratory time; tPTEF_SLP_, SLP‐derived time to reach peak tidal expiratory flow; tPTIF_SLP_, SLP‐derived time to reach peak tidal inspiratory flow; v, within‐subject variability.

**Figure 4:**
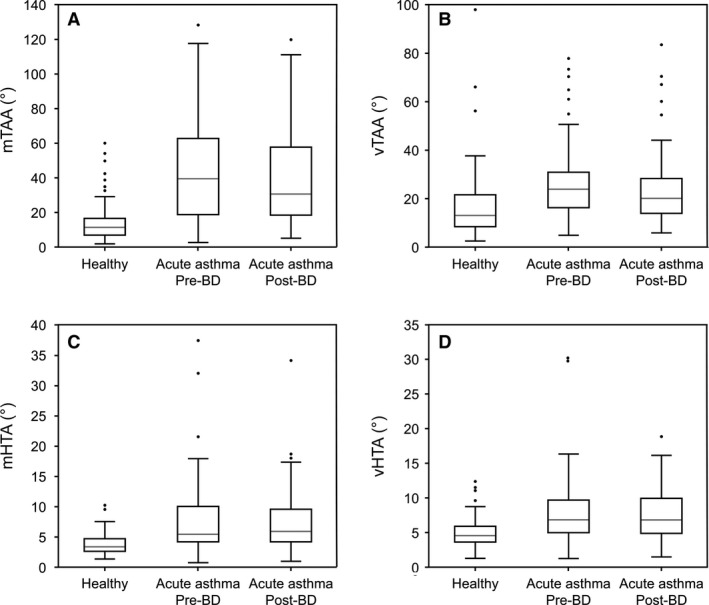
The asynchrony‐based parameters mTAA (A), vTAA (B), mHTA (C), and vHTA (D) differed in healthy children (*n* = 54) compared with those with asthma/wheeze (*n* = 39) and remained so after bronchodilator administration. The gray line indicates the median value, the rectangle spans the interquartile range, and the black whiskers indicate the minimum and maximum values (excluding the outliers indicated by the black circles). BD, bronchodilator; HTA, left–right hemi‐thoracic asynchrony; m, median; SLP, structured light plethysmography; TAA, thoraco–abdominal asynchrony; v, within‐subject variability.

**Figure 5:**
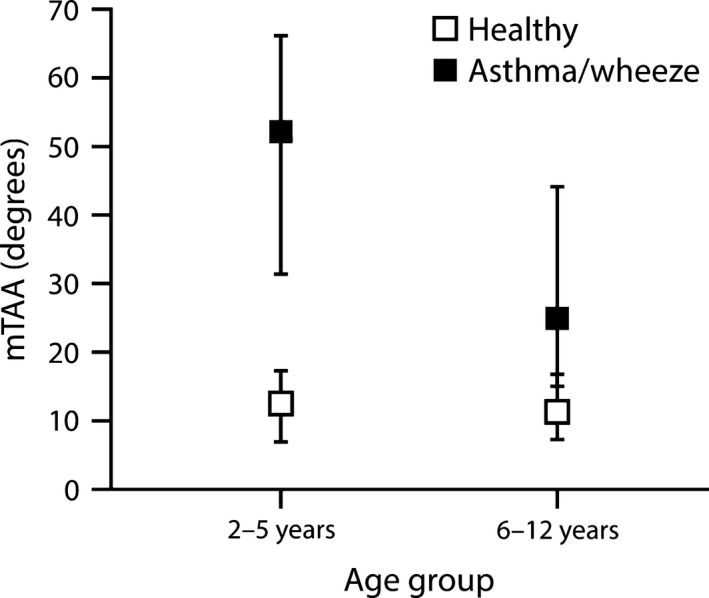
mTAA in healthy children and those with asthma/wheeze, stratified by age group. Error bars indicate the 25th and 75th quartiles. m, median; TAA, thoraco–abdominal asynchrony.

Several parameters differed significantly between children recovering from an acute exacerbation of asthma/wheeze (before bronchodilator administration) and healthy controls (Table 2). Of the timing parameters, mRR was significantly higher in children with asthma (30 vs. 23 brpm; *P* < 0.001), while mtI (0.83 vs. 1.13 sec), mtE (1.14 vs. 1.48 sec), and mtTot (2.00 vs. 2.60 sec) were lower (all *P* < 0.001). With the exception of vRR, within‐subject variability in all timing indices and ratios were significantly lower in children with asthma/wheeze than in healthy controls. Of the flow‐based parameters, mIE50_SLP_ was significantly higher (1.47 vs. 1.31, *P* = 0.002), while the within‐subject variability in tPTIF_SLP_/tI was significantly lower in children with asthma/wheeze compared with healthy children (0.16 vs. 0.21, *P* < 0.001; Fig. 3). Both asynchrony parameters (mTAA and mHTA) were significantly higher in children with asthma/wheeze (mTAA: 40.16 vs. 11.88°; mHTA: 5.53 vs. 3.43°; both *P* < 0.001; Fig. 4), as were the variability in both these parameters (vTAA: 24.08 vs. 13.53°; vHTA, 6.82 vs. 4.58°; both *P* < 0.001; Fig. 4). The effects of asthma/wheeze on younger children (aged 2–5 years) differed from those on older children (aged 6–12 years) for mTAA only (interaction *P* < 0.001; Table 2). For healthy children, mTAA decreased slightly with age (12.6 and 11.4° for younger and older children, respectively), but for children with asthma/wheeze, mTAA decreased by more than 50% from 52.2° in the younger cohort to 25.1° in the older cohort (Fig. 5).

**Table 2:** SLP‐assessed tidal breathing parameters in children with acute asthma/wheeze (before bronchodilator administration) versus healthy children. Significantly different parameters are shown in bold italics

	Healthy children (*N* = 54)		Children with acute asthma/wheeze (before bronchodilator) (*N* = 39)			Overall significance (MWU test)	Age group interaction significance^a^ (robust ANOVA)
	Median	IQR	Median	IQR	*z*‐statistic	*P*‐value	*P*‐value
Timing indices and ratios							
***mRR (brpm)***	***23.00***	***20.00***–***25.35***	***30.00***	***24.87***–***32.58***	−***4.74***	**<** ***0.001*** c	0.369
vRR (brpm)	4.57	3.39–6.34	4.45	3.33–6.49	0.11	0.913	0.761
***mtI (sec)***	***1.13***	***0.96***–***1.26***	***0.83***	***0.80***–***0.99***	***5.44***	**<** ***0.001*** c	0.569
***vtI (sec)***	***0.22***	***0.16***–***0.36***	***0.13***	***0.09***–***0.21***	***3.99***	**<** ***0.001*** c	0.476
***mtE (sec)***	***1.48***	***1.33***–***1.73***	***1.14***	***0.98***–***1.41***	***4.10***	**<** ***0.001*** c	0.888
***vtE (sec)***	***0.43***	***0.30***–***0.55***	***0.23***	***0.17***–***0.32***	***4.87***	**<** ***0.001*** c	0.385
***mtTot (sec)***	***2.60***	***2.36***–***3.00***	***2.00***	***1.84***–***2.41***	***4.74***	**<** ***0.001*** c	0.727
***vtTot (sec)***	***0.53***	***0.41***–***0.72***	***0.33***	***0.26***–***0.37***	***4.86***	**<** ***0.001*** c	1.000
mtI/tE	0.73	0.68–0.81	0.70	0.64–0.79	1.20	0.229	0.653
***vtI/tE***	***0.23***	***0.18***–***0.30***	***0.16***	***0.13***–***0.21***	***3.55***	**<** ***0.001*** b	0.397
mtI/tTot	0.42	0.40–0.44	0.41	0.39–0.44	1.20	0.229	0.652
***vtI/tTot***	***0.07***	***0.06***–***0.09***	***0.05***	***0.04***–***0.07***	***3.37***	***0.001*** c	0.248
Flow‐based parameters							
mtPTEF_SLP_/tE	0.34	0.28–0.39	0.38	0.29–0.47	−1.76	0.079	0.987
vtPTEF_SLP_/tE	0.22	0.16–0.26	0.21	0.13–0.33	0.14	0.885	0.102
mtPTIF_SLP_/tI	0.55	0.50–0.60	0.53	0.50–0.56	1.18	0.236	0.248
***vtPTIF***_***SLP***_ ***/tI***	***0.21***	***0.18***–***0.27***	***0.16***	***0.13***–***0.19***	***4.65***	**<** ***0.001*** c	0.113
***mIE50***_***SLP***_	***1.31***	***1.20***–***1.50***	***1.47***	***1.33***–***1.73***	−***3.13***	***0.002*** b	0.335
vIE50_SLP_	0.60	0.49–0.82	0.56	0.39–0.80	1.01	0.313	0.130
Regional parameters (relative contribution and asynchrony)							
mrCT (%)	41.01	33.97–48.45	42.86	33.96–54.65	−0.77	0.439	0.876
vrCT (%)	9.22	6.17–13.00	10.13	6.54–13.94	−0.60	0.551	0.271
***mHTA (°)***	***3.43***	***2.63***–***4.72***	***5.53***	***4.18***–***9.97***	−***4.47***	**<** ***0.001*** c	0.566
***vHTA (°)***	***4.58***	***3.68***–***5.87***	***6.82***	***5.04***–***9.71***	−***3.64***	**<** ***0.001*** c	0.550
***mTAA (°)***	***11.88***	***7.23***–***17.07***	***40.16***	***19.12***–***62.67***	−***5.41***	**<** ***0.001*** c	**<** ***0.001*** c
***vTAA (°)***	***13.53***	***8.80***–***21.77***	***24.08***	***16.57***–***31.28***	−***4.21***	**<** ***0.001*** c	0.170
***Number of breaths***	***81***	***65***–***92***	***103***	***84.5***–***120***	−***4.11***	**<** ***0.001*** c	0.269

Median values (denoted by “m”) for all tidal breathing parameters were calculated for each participant, in addition to its IQR as a measure of the within‐subject variability (denoted by “v”). Individual data for all participants in each cohort were then combined and are summarized in the table by their median and IQR.

ANOVA, analysis of variance; brpm, breaths per minute; HTA, left–right hemi‐thoracic asynchrony; IE50_SLP_, SLP‐derived tidal inspiratory flow at 50% of inspiratory volume divided by tidal expiratory flow at 50% of expiratory volume; IQR, interquartile range; MWU, Mann–Whitney *U*; rCT, relative contribution of the thorax to each breath; RR, respiratory rate; SLP, structured light plethysmography; TAA, thoraco–abdominal asynchrony; tE, expiratory time; tI, inspiratory time; tPTEF_SLP_, SLP‐derived time to reach peak tidal expiratory flow; tPTIF_SLP_, SLP‐derived time to reach peak tidal inspiratory flow; tTot, total breath time.

a A robust ANOVA was used to determine whether differences in effect of asthma/wheeze on tidal breathing parameters differed between younger (aged 2–5 years) and older (aged 6–12 years) children.

b Significant with *P* < 0.01.

c Significant with *P *< 0.001. All tests of overall significance had 69 degrees of freedom.

**Figure 6:**
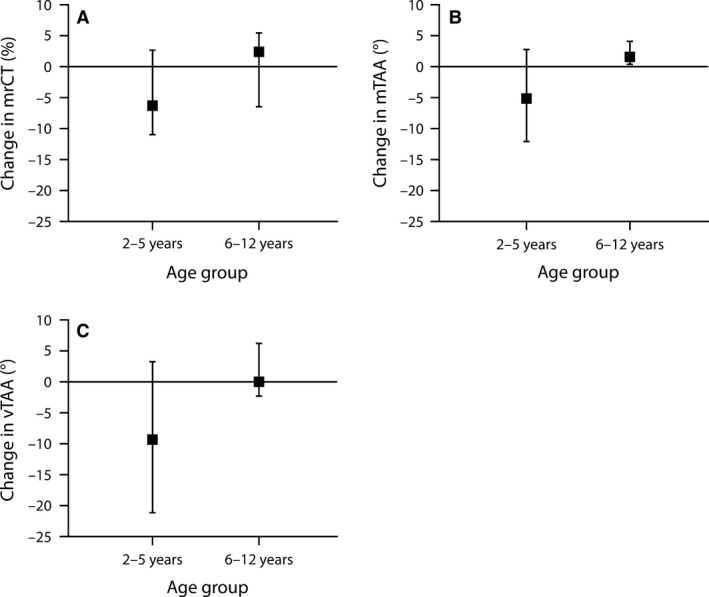
Change in (A) mrCT, (B) mTAA, and (C) vTAA after treatment with bronchodilator in children with asthma/wheeze, stratified by age group. Error bars indicate the 25th and 75th quartiles. m, median; rCT, relative contribution of the thorax; TAA, thoraco–abdominal asynchrony; v, within‐subject variability.

No median parameter changed significantly after bronchodilator administration for the overall asthma/wheeze cohort (Table 3). The only change observed was the within‐subject variability in tPTEF_SLP_/tE, which decreased from 0.21 to 0.15 (*P* < 0.001; Fig. 3). When assessed according to age, the older and younger cohorts differed in the effects of bronchodilator administration for mrCT, mTAA, and vTAA (interaction *P* < 0.05). Median rCT decreased significantly in the younger cohort after bronchodilator administration (interaction *P* < 0.05 and WSR *P* < 0.05), but did not change significantly in older children and in fact increased for most individuals in this cohort (Fig. 6). The effects of bronchodilator administration on mTAA were also significantly different in the two cohorts, with asynchrony decreasing in younger children but increasing in the older cohort, although the effects were not significantly different from zero in either age group (Fig. 6). Similarly, vTAA significantly decreased in the younger cohort following bronchodilator treatment (*P* < 0.05), but did not change in the older cohort (Fig. 6). All parameters that were significantly different between healthy children and those with asthma/wheeze before administration of the bronchodilator remained so after (Table 4).

**Table 3:** SLP‐assessed tidal breathing parameters in children with acute asthma/wheeze before and after bronchodilator administration. Significantly different parameters are shown in bold italics

	Children with acute asthma/wheeze (before bronchodilator) (*N* = 39)		Children with acute asthma/wheeze (after bronchodilator) (*N* = 39)			Overall significance (WSR test)	Age group interaction significance^a^ (MWU test)
	Median	IQR	Median	IQR	*z*‐statistic	*P*‐value	*P*‐value
Timing indices and ratios							
mRR (brpm)	30.00	24.87–32.58	31.03	25.08–33.33	−1.56	0.118	0.305
vRR (brpm)	4.45	3.33–6.49	4.36	3.73–6.58	−0.47	0.635	0.146
mtI (sec)	0.83	0.80–0.99	0.80	0.74–0.95	1.61	0.108	0.612
vtI (sec)	0.13	0.09–0.21	0.13	0.09–0.20	0.50	0.619	0.828
mtE (sec)	1.14	0.98–1.41	1.13	1.00–1.40	1.06	0.290	0.175
vtE (sec)	0.23	0.17–0.32	0.25	0.19–0.34	−0.82	0.410	0.603
mtTot (sec)	2.00	1.84–2.41	1.93	1.80–2.39	1.61	0.107	0.363
vtTot (sec)	0.33	0.26–0.37	0.32	0.23–0.43	−0.30	0.763	0.419
mtI/tE	0.70	0.64–0.79	0.70	0.62–0.76	1.03	0.301	0.665
vtI/tE	0.16	0.13–0.21	0.14	0.13–0.19	1.35	0.176	0.283
mtI/tTot	0.41	0.39–0.44	0.41	0.38–0.43	0.97	0.331	0.707
vtI/tTot	0.05	0.04–0.07	0.05	0.04–0.06	1.31	0.190	0.246
Flow‐based parameters							
mtPTEF_SLP_/tE	0.38	0.29–0.47	0.37	0.31–0.45	0.85	0.395	0.679
***vtPTEF***_***SLP***_ ***/tE***	***0.21***	**0.13–0.33**	***0.15***	**0.11–0.23**	***3.87***	**<** ***0.001*** c	0.352
mtPTIF_SLP_/tI	0.53	0.50–0.56	0.53	0.50–0.56	−1.24	0.213	0.564
vtPTIF_SLP_/tI	0.16	0.13–0.19	0.17	0.12–0.20	0.10	0.922	0.658
mIE50_SLP_	1.47	1.33–1.73	1.50	1.35–1.67	0.71	0.477	0.598
vIE50_SLP_	0.56	0.39–0.80	0.52	0.37–0.74	1.84	0.065	0.309
Regional parameters (relative contribution and phase)							
mrCT (%)	42.86	33.96–54.65	39.47	31.34–51.19	1.95	0.051	**0.041** b
vrCT (%)	10.13	6.54–13.94	8.98	6.48–11.06	1.45	0.147	0.051
mHTA (°)	5.53	4.18–9.97	5.98	4.18–9.51	0.82	0.41	0.338
vHTA (°)	6.82	5.04–9.71	6.82	4.84–9.93	0.03	0.978	0.449
mTAA (°)	40.16	19.12–62.67	31.08	18.63–57.89	0.89	0.372	**0.030** b
vTAA (°)	24.08	16.57–31.28	20.31	14.14–28.71	1.41	0.159	**0.020** b
Number of breaths	103	84.5–120	107	93–115.8	−1.68	0.094	0.862

Median values (denoted by “m”) for all tidal breathing parameters were calculated for each participant, in addition to its IQR as a measure of the within‐subject variability (denoted by “v”). Individual data for all participants in each cohort were then combined and are summarized in the table by their median and IQR.

brpm, breaths per minute; HTA, left–right hemi‐thoracic asynchrony; IE50_SLP_, SLP‐derived tidal inspiratory flow at 50% of inspiratory volume divided by tidal expiratory flow at 50% of expiratory volume; IQR, interquartile range; MWU, Mann‐Whitney *U*; rCT, relative contribution of the thorax to each breath; RR, respiratory rate; SLP, structured light plethysmography; TAA, thoraco–abdominal asynchrony; tE, expiratory time; tI, inspiratory time; tPTEF_SLP_, SLP‐derived time to reach peak tidal expiratory flow; tPTIF_SLP_, SLP‐derived time to reach peak tidal inspiratory flow; tTot, total breath time; WSR, Wilcoxon signed‐rank.

a A MWU test of the differences before and after bronchodilator was used to determine whether the effects of bronchodilator on tidal breathing parameters differed between younger (aged 2–5 years) and older (aged 6–12 years) children.

b Significant with *P *< 0.05.

c Significant with *P *< 0.001. All tests of overall significance had 69 degrees of freedom.

**Table 4:** SLP‐assessed tidal breathing parameters^a^ in children with acute asthma/wheeze (after bronchodilator administration) versus healthy children. Significantly different parameters are shown in bold italics

	Healthy children (*N* = 54)		Children with acute asthma/wheeze (after bronchodilator) (*N* = 39)			Overall significance (MWU test)	Age group interaction significance^b^ (robust ANOVA)
	Median	IQR	Median	IQR	*z*‐statistic	*P*‐value	*P*‐value
Timing indices and ratios							
***mRR (brpm)***	***23.00***	***20.00***–***25.35***	***31.03***	***25.08***–***33.33***	−***5.01***	**<** ***0.001*** d	0.642
***mtI (sec)***	***1.13***	***0.96*** –***1.26***	***0.8***	***0.74***–***0.95***	***5.67***	**<** ***0.001*** d	1.000
***vtI (sec)***	***0.22***	***0.16***–***0.36***	***0.13***	***0.09***–***0.20***	***4.57***	**<** ***0.001*** d	0.782
***mtE (sec)***	***1.48***	***1.33***–***1.73***	***1.13***	***1.00***–***1.40***	***4.17***	**<** ***0.001*** d	0.814
***vtE (sec)***	***0.43***	***0.30***–***0.55***	***0.25***	***0.19***–***0.34***	***4.72***	**<** ***0.001*** d	0.195
***mtTot (sec)***	***2.60***	***2.36***–***3.00***	***1.93***	***1.80***–***2.39***	***5.02***	**<** ***0.001*** d	0.924
***vtTot (sec)***	***0.53***	***0.41***–***0.72***	***0.32***	***0.23***–***0.43***	***4.66***	**<** ***0.001*** d	0.508
***vtI/tE***	***0.23***	***0.18***–***0.30***	***0.14***	***0.13***–***0.19***	***4.18***	**<** ***0.001*** d	0.663
***vtI/tTot***	***0.07***	***0.06***–***0.09***	***0.05***	***0.04***–***0.06***	***4.09***	**<** ***0.001*** d	0.321
Flow‐based parameters							
***vtPTIF***_***SLP***_ ***/tI***	***0.21***	***0.18***–***0.27***	***0.17***	***0.12***–***0.20***	***4.26***	**<** ***0.001*** d	0.083
***mIE50***_***SLP***_	***1.31***	***1.20***–***1.50***	***1.50***	***1.35***–***1.67***	−***3.26***	***0.001*** c	0.350
Regional parameters (relative contribution and phase)							
***mHTA (°)***	***3.43***	***2.63***–***4.72***	***5.98***	***4.18***–***9.51***	−***4.11***	**<** ***0.001*** d	0.796
***vHTA (°)***	***4.58***	***3.68***–***5.87***	***6.82***	***4.84***–***9.93***	−***3.29***	***0.001*** c	0.767
***mTAA (°)***	***11.88***	***7.23***–***17.07***	***31.08***	***18.63***–***57.89***	−***5.21***	**<** ***0.001*** d	0.054
***vTAA (°)***	***13.53***	***8.80***–***21.77***	***20.31***	***14.14***–***28.71***	−***3.34***	***0.001*** c	0.682
***Number of breaths***	***81***	***65***–***92***	***107***	***93***–***115.8***	−***5.33***	**<** ***0.001*** d	0.271

Median values (denoted by “m”) for all tidal breathing parameters were calculated for each participant, in addition to its IQR as a measure of the within‐subject variability (denoted by “v”). Individual data for all participants in each cohort were then combined and are summarized in the table by their median and IQR.

ANOVA, analysis of variance; brpm, breaths per minute; HTA, left–right hemi‐thoracic asynchrony; IE50_SLP_, SLP‐derived tidal inspiratory flow at 50% of inspiratory volume divided by tidal expiratory flow at 50% of expiratory volume; IQR, interquartile range; MWU, Mann–Whitney *U*; RR, respiratory rate; SLP, structured light plethysmography; TAA, thoraco–abdominal asynchrony; tE, expiratory time; tI, inspiratory time; tPTIF_SLP_, SLP‐derived time to reach peak tidal inspiratory flow; tTot, total breath time.

a Data are shown only for those parameters that differed between children with asthma (before bronchodilator administration) and healthy children (see Table 2).

b A robust ANOVA was used to determine whether differences in effect of asthma/wheeze on tidal breathing parameters differed between younger (aged 2–5 years) and older (aged 6–12 years) children.

c Significant with *P* < 0.01.

d Significant with *P *< 0.001. All tests of overall significance had 69 degrees of freedom.

According to CLES evaluation, mtI and mTAA demonstrated the largest effect in distinguishing healthy children from those with acute asthma (Table 5). These parameters also showed the largest effect size in distinguishing the acute asthma group from normal after bronchodilator administration (mtI: 83.4%; mTAA: 81.8%), in addition to mRR and mtTot (both 80.2%). Furthermore, in children with asthma, within‐subject variability in tPTEF_SLP_/tE could detect bronchodilator effects in 74.4% of cases (Table 5).

**Table 5:** CLES evaluation of SLP‐obtained breathing parameters

Hypothesis	CLES (%)	Interpretation
Healthy vs. children with asthma^a^ (before BD administration)		
mRR: higher in asthma group	78.5	In 78.5% of cases, mRR was higher in asthma group
mtI: lower in asthma group	82.1	In 82.1% of cases, mtI was lower in asthma group
vtI: lower in asthma group	73.2	In 73.2% of cases, vtI was lower in asthma group
mtE: lower in asthma group	74.2	In 74.2% of cases, mtE was lower in asthma group
vtE: lower in asthma group	79.2	In 79.2% of cases, vtE was lower in asthma group
mtTot: lower in asthma group	78.5	In 78.5% of cases, mtTot was lower in asthma group
vtTot: lower in asthma group	79.1	In 79.1% of cases, vtTot was lower in asthma group
vtI/tE: lower in asthma group	71.7	In 71.7% of cases, vtI/tE was lower in asthma group
vtI/tTot: lower in asthma group	70.6	In 70.6% of cases, vtI/tTot was lower in asthma group
vtPTIF_SLP_/tI: lower in asthma group	78.4	In 78.4% of cases, vtPTIF_SLP_/tI was lower in asthma group
mIE50_SLP_: higher in asthma group	69.1	In 69.1% of cases, mIE50_SLP_ was higher in asthma group
mHTA: higher in asthma group	77.3	In 77.3% of cases, mHTA was higher in asthma group
vHTA: higher in asthma group	72.2	In 72.2% of cases, vHTA was higher in asthma group
mTAA: higher in asthma group	83.0	In 83.0% of cases, mTAA was higher in asthma group
vTAA: higher in asthma group	75.7	In 75.7% of cases, vTAA was higher in asthma group
Healthy vs. children with asthma^a^ (after BD administration)		
mRR: higher in asthma group	80.2	In 80.2% of cases, mRR was higher in asthma group
mtI: lower in asthma group	83.4	In 83.4% of cases, mtI was lower in asthma group
vtI: lower in asthma group	76.5	In 76.5% of cases, vtI was lower in asthma group
mtE: lower in asthma group	74.9	In 74.9% of cases, mtE was lower in asthma group
vtE: lower in asthma group	78.1	In 78.1% of cases, vtE was lower in asthma group
mtTot: lower in asthma group	80.2	In 80.2% of cases, mtTot was lower in asthma group
vtTot: lower in asthma group	77.9	In 77.9% of cases, vtTot was lower in asthma group
vtI/tE: lower in asthma group	75.5	In 75.5% of cases, vtI/tE was lower in asthma group
vtI/tTot: lower in asthma group	75.0	In 75.0% of cases, vtI/tTot was lower in asthma group
vtPTEF_SLP_/tE: lower in asthma group	64.9	In 64.9% of cases, vtPTEF_SLP_/tE was lower in asthma group
vtPTIF_SLP_/tI: lower in asthma group	76.0	In 76.0% of cases, vtPTIF_SLP_/tI was lower in asthma group
mIE50_SLP_: higher in asthma group	69.9	In 69.9% of cases, mIE50_SLP_ was higher in asthma group
mHTA: higher in asthma group	75.1	In 75.1% of cases, mHTA was higher in asthma group
vHTA: higher in asthma group	70.1	In 70.1% of cases, vHTA was higher in asthma group
mTAA: higher in asthma group	81.8	In 81.8% of cases, mTAA was higher in asthma group
vTAA: higher in asthma group	70.4	In 70.4% of cases, vTAA was higher in asthma group
Before vs. after BD administration^b^ (children with asthma)		
vtPTEF_SLP_/tE: reduced after BD	74.4	In 74.4% of cases, vtPTEF_SLP_/tE decreased after BD

Median and interquartile range values for each parameter are denoted by the prefix “m” and “v”, respectively.

BD, bronchodilator; CLES, common language effect size; HTA, left–right hemi‐thoracic asynchrony; IE50_SLP_, SLP‐derived tidal inspiratory flow at 50% of inspiratory volume divided by expiratory flow at 50% of expiratory volume; RR, respiratory rate; SLP, structured light plethysmography; TAA, thoraco–abdominal asynchrony; tE, expiratory time; tI, inspiratory time; tPTEF_SLP_, SLP‐derived time to reach peak tidal expiratory flow; tPTIF_SLP_, SLP‐derived time to reach peak tidal inspiratory flow; tTot, total breath time.

a Data are shown for parameters that significantly differed between healthy children and children with asthma (pre‐ and post‐bronchodilator administration) only (see Tables 2 and 4).

b Data are shown for parameters that significantly differed following BD administration in children with asthma only (see Table 3).

## Discussion

We compared SLP‐assessed tidal breathing parameters in children aged 2–12 years who were recovering from an acute exacerbation of asthma/wheeze and had received bronchodilator intervention in the course of their treatment with those of healthy children of the same age. We also carried out a secondary analysis to examine whether the effect of asthma/wheeze or the effect of administration of a bronchodilator differed between younger (aged 2–5 years) and older (aged 6–12 years) children. In the overall cohort, median values of seven parameters, and the within‐subject variability of eight parameters, were identified that differed between children with and without acute asthma/wheeze. After a further bronchodilator administration, no change was observed in the median value of any parameter; however, a reduction was observed in the within‐subject variability of one flow‐based parameter. We did, however, observe that the response to both asthma/wheeze and to bronchodilation differed between younger and older children, with greater changes seen in regional parameters (TAA and rCT) in younger children.

In the overall cohort, median IE50_SLP_ was higher in children with acute asthma/wheeze than those in the healthy group. The conventional tidal breathing parameter IE50 is defined as the ratio of inspiratory to expiratory flow at 50% of tidal volume (Stick 1996). Studies have demonstrated a reduction in TEF50 in obstructive airway disorders including asthma (Totapally et al. 1996; Papiris et al. 2002; Tauber et al. 2003). A reduction in TEF50, without a reduction in TIF50, would increase IE50 and explain the higher median IE50_SLP_ observed in our study. Elevated IE50 has been reported in other populations, including in our previous studies in children aged 7–16 years with nonacute asthma (Hmeidi et al. 2017) and adults with COPD (Motamedi‐Fakhr et al. 2017b). IE50_SLP_ did not respond to the additional bronchodilator treatment administered to children with acute asthma/wheeze during this study and remained higher than normal despite the children being in the recovery phase of their illness and considered clinically stable. This is in contrast to our findings in children with nonacute asthma where a significant decrease in IE50_SLP_ was observed following bronchodilator administration (Hmeidi et al. 2017). In this previous study, it was known that the children had a lower forced expiratory volume in 1 sec (FEV_1_) prior to the bronchodilator intervention and that the reduction in IE50_SLP_ following bronchodilator treatment was associated with an increase in % predicted FEV_1,_ indicating a bronchodilator response. In the present study, however, it was unknown whether FEV1 was low before bronchodilator intervention as spirometry was not performed. Therefore, it may be possible that the increased IE50_SLP_ observed was indicative of the component of airflow obstruction that is insensitive to bronchodilator, or that there was simply no bronchodilator response to observe. It is of note that in the previous study in children with nonacute asthma, IE50_SLP_ remained significantly higher than normal after bronchodilation (Hmeidi et al. 2017). Our observations in the present study may suggest that, although considered in the recovery phase, these children were still experiencing the effects of respiratory exacerbation. In other studies, bronchodilator treatment in patients with asthma was followed by a return of traditional tidal breathing parameters toward normal (Kuratomi et al. 1985; van der Ent et al. 1996). It would be of interest to directly compare IE50_SLP_ in the same asthmatic children with and without an exacerbation and throughout recovery from an exacerbation in order to determine whether this variable could be used to monitor disease activity.

Both asynchrony parameters (TAA and HTA) were significantly greater in children with acute asthma/wheeze compared with healthy controls, as was their within‐subject variability. Although some asynchrony can be detected in healthy children (Sivan et al. 1990; Newth and Hammer 2005), generally the thorax and abdomen move in phase in those without obstructive disease. However, when the work of breathing increases in children with acute asthma, movement of the abdomen precedes that of the thorax, resulting in a loss of this synchrony (Carlsen and Lodrup Carlsen 2010; Giordano et al. 2012). The observation that within‐subject variability in asynchrony is greater in children with asthma both between the thorax and abdomen and between the left and right hemi‐thorax may suggest a compensatory mechanism in which spatial variability is introduced into the system when temporal variability is reduced. Within‐subject variability of asynchrony was not previously observed in children with nonacute asthma when compared with healthy subjects (Hmeidi et al. 2017); however, the children in that study were older so were likely to have reduced chest wall compliance, and thus, less propensity for regional variation. This effect of age is further supported by our observation in the current study that the effect of asthma/wheeze on TAA was greater in younger children than in the older cohort. To our knowledge, the effects of acute asthma on HTA (or on variability in asynchrony parameters) have not been reported before.

In contrast to the increased within‐subject variability observed in asynchrony parameters, variability of tPTIF_SLP_/tI was lower than normal in the acute asthma group. This was not as we had expected as it has previously been reported that children with asthma have greater variability in, for example, airway resistance (Lall et al. 2007). Our observation may have been attributable to the repeated bronchodilator treatment received by our patient group prior to the test intervention. The variability in tPTEF_SLP_/tE reduced in response to bronchodilator treatment, which is in accordance with that reported for the variation in airways resistance in both asthmatics and controls following administration of a bronchodilator (Lall et al. 2007). Similarly, in our previous study in children with nonacute asthma, we detected a nonsignificant reduction in the variation of tPTEF_SLP_/tE in response to bronchodilator intervention, and the difference observed between healthy and asthmatics pre‐bronchodilator was no longer apparent post‐bronchodilator, suggesting that some reduction had occurred (Hmeidi et al. 2017). No such reduction was observed in the variability of IE50_SLP_ in response to bronchodilator treatment in our group of patients recovering from an acute exacerbation. In the study by Lall et al. (2007), it was reported that reduction in variability in airways resistance exceeded that of FEV1. Our observations may suggest that the variability of tPTEF/tE may, similarly, be more sensitive to the effects of bronchodilator intervention than the variability of IE50. Further work will be required to investigate this.

Compared with older children, administration of bronchodilator had a greater effect on mTAA and vTAA in younger children, who exhibited reduced and less variable asynchrony. Furthermore, their breathing also became more abdominal as indicated by reduced mrCT. These observations had not been apparent in our previous study of older children, so we suggest that it is a characteristic effect in younger children due to differences in chest wall compliance.

In the present study, RR was higher in acute asthma/wheeze and the duration of the respiratory cycle as a whole (i.e., tTot) and its components (tI and tE) were shorter compared with those of healthy children. Patients with acute asthma have a higher RR than normal to compensate for the reduced amount of air inhaled at each breath as a result of airway obstruction (Kesten et al. 1990). With the exception of RR, all timing indices and ratios showed reduced within‐subject variability in children with acute asthma/wheeze. This decrease was expected as the RR was faster in these children, allowing less freedom for variation. As observed in our study, healthy subjects typically display some variability in tidal breathing parameters (Tobin et al. 1988). The propensity for normal breathing patterns to vary allows the respiratory system to participate in tasks besides gas exchange, such as speech and coughing (Brack et al. 2002). SLP is well placed to assess within‐subject variability as it involves the measurement of a large number of consecutive breaths (mean ≥80 breaths per assessment in the current study).

SLP is a noncontact technique that does not require equipment such as facemasks that may inadvertently influence tidal breathing, and requires only minimal cooperation from the subject. One limitation of the method is that it requires individuals to stay still for several minutes. Consequently, we did not attempt to assess children with asthma who presented with an acute exacerbation until they were in the recovery phase of the illness and considered clinically stable. It is likely, therefore, that the study missed changes in tidal breathing parameters occurring during the exacerbation. Furthermore, assessment of the SLP response to bronchodilators was confounded by the previous bronchodilator treatments received since admission and prior to enrollment.

As multiple comparisons were made during this study, the risk of some statistically significant results occurring by chance should be considered. The Bonferroni correction was not applied as this method assumes that all comparisons are independent, which was not the case here. Initial statistical comparisons were supported by CLES evaluation, and many of the observed changes in SLP parameters appear to have a firm physiological basis or are corroborated by other studies (Laveneziana et al. 2015b; Motamedi‐Fakhr et al. 2017b).

Here, we have shown that SLP can be performed successfully in children as young as 2 years of age recovering from acute asthma/wheeze. In addition, certain SLP parameters, in particular IE50_SLP_, RR and asynchrony (both hemi‐thoracic and thoraco–abdominal), along with the within‐subject variability of multiple parameters, differed in the acute asthma group, and so may offer the clinician a means of distinguishing between these children and their healthy counterparts, and also a means of monitoring recovery. SLP may prove particularly useful in the preschool age group where providing an accurate asthma diagnosis is a major clinical challenge due to the difficulties in assessing airflow limitation at this age. These preliminary results look promising and support further study and refinement of the technique and data analysis methods with an aim toward introduction into routine clinical practice. Further study is also necessary to evaluate the effects of age on breathing patterns; SLP may represent a method for assessing lung function in patient populations in whom traditional techniques such as spirometry cannot be conveniently used.

## Conflicts of Interest

SMF and RCW are employees of and have share options for PneumaCare Ltd. (Ely, Cambridgeshire, UK). EKC received funding for a PhD student (HH) under his supervision from PneumaCare Ltd. WL is employed part‐time as a pediatric respiratory advisor to GSK. RI is a shareholder of and part‐time paid medical advisor to PneumaCare Ltd. HH, JA, and FJG have declared no conflicts of interest, financial, or otherwise.

## References

[phy213752-bib-0001] Bates, J. H. , G. Schmalisch , D. Filbrun , J. Stocks . 2000 Tidal breath analysis for infant pulmonary function testing. ERS/ATS Task Force on standards for infant respiratory function testing. Eur. Respir. J. 16:1180–1192. 11292125 10.1034/j.1399-3003.2000.16f26.x

[phy213752-bib-0002] Beydon, N. , S. D. Davis , E. Lombardi , J. L. Allen , H. G. Arets , P. Aurora , et al. 2007 An official American Thoracic Society/European Respiratory Society statement: pulmonary function testing in preschool children. Am. J. Respir. Crit. Care Med. 175:1304–1345. 17545458 10.1164/rccm.200605-642ST

[phy213752-bib-0003] Brack, T. , A. Jubran , and M. J. Tobin . 2002 Dyspnea and decreased variability of breathing in patients with restrictive lung disease. Am. J. Respir. Crit. Care Med. 165:1260–1264. 11991875 10.1164/rccm.2201018

[phy213752-bib-0004] Caretti, D. M. , P. V. Pullen , L. A. Premo , W. D. Kuhlmann . 1994 Reliability of respiratory inductive plethysmography for measuring tidal volume during exercise. Am. Ind. Hyg. Assoc. J. 55:918–923. 7977031 10.1080/15428119491018411

[phy213752-bib-0005] Carlsen, K. , and K. Lodrup Carlsen . 2010 Tidal breathing techniques Pp. 35–45 *in* Frey U., Merkus P., eds. European Respiratory Monograph 47: paediatric lung function. European Respiratory Society, Sheffield, UK.

[phy213752-bib-0006] van der Ent, C. K. , H. J. Brackel , J. van der Laag , J. M Bogaard . 1996 Tidal breathing analysis as a measure of airway obstruction in children three years of age and older. Am. J. Respir. Crit. Care Med. 153:1253–1258. 8616550 10.1164/ajrccm.153.4.8616550

[phy213752-bib-0007] Giordano, K. , E. Rodriguez , N. Green , M. Armani , J. Richards , T. H. Shaffer , et al. 2012 Pulmonary function tests in emergency department pediatric patients with acute wheezing/asthma exacerbation. Pulm. Med. 2012:724139. 23304496 10.1155/2012/724139 PMC3523566

[phy213752-bib-0008] Global Initiative for Chronic Obstructive Lung Disease (GOLD). 2010 Spirometry for health care providers 2010. Available at: http://goldcopd.org/wp-content/uploads/2016/04/GOLD_Spirometry_2010.pdf [Last accessed 02 January 2018].

[phy213752-bib-0010] Hmeidi, H. , S. Mortamedi‐Fakhr , E. Chadwick , W. Lenney , R. Iles , R. C. Wilson , et al. 2017 Tidal breathing parameters measured using structured light plethysmography in healthy children and those with asthma before and after bronchodilator. Physiol. Rep. 5:e13168. 28275111 10.14814/phy2.13168 PMC5350176

[phy213752-bib-0011] Kesten, S. , M. R. Maleki‐Yazdi , B. R. Sanders , J. A. Wells , S. L. McKillop , K. R. Chapman , et al. 1990 Respiratory rate during acute asthma. Chest 97:58–62. 2403901 10.1378/chest.97.1.58

[phy213752-bib-0012] Kloke, J. D. , and J. W. McKean . 2012 Rfit: rank‐based estimation for linear models. R J. 4:57–64.

[phy213752-bib-0013] Kuratomi, Y. , N. Okazaki , T. Ishihara , T. Arai , S. Kira . 1985 Variability of breath‐by‐breath tidal volume and its characteristics in normal and diseased subjects. Ventilatory monitoring with electrical impedance pneumography. Jpn. J. Med. 24:141–149. 4021211 10.2169/internalmedicine1962.24.141

[phy213752-bib-0014] Lall, C. A. , N. Cheng , P. Hernandez , P. T. Pianosi , Z. Dali , A. Abouzied , et al. 2007 Airway resistance variability and response to bronchodilator in children with asthma. Eur. Respir. J. 30:260–268. 17331970 10.1183/09031936.00064006

[phy213752-bib-0015] Laveneziana, P. , C. Llontop , M. C. Nierat , A. Bellocq , C. Straus , and T. Similowski . 2015a Disruption of tidal breathing in COPD by use of pneumotachograph and mouthpiece compared to non‐contact measurement with structured light plethysmography (SLP). Eur. Respir. J. 46:PA511.

[phy213752-bib-0016] Laveneziana, P. , C. Llontop , M.‐C. Nierat , A. Bellocq , C. Straus , and T. Similowski . 2015b Non‐contact assessment of acute bronchodilator’ response during tidal breathing in COPD patients using structured light plethysmography (SLP). Eur. Respir. J. 46:A2270.

[phy213752-bib-0017] Motamedi‐Fakhr, S. , R. Iles , A. Barney , W. Boer , J. Conlon , A. Khalid , et al. 2017a Evaluation of the agreement of tidal breathing parameters measured simultaneously using pneumotachography and structured light plethysmography. Physiol. Rep. 5:e13124. 28193785 10.14814/phy2.13124 PMC5309576

[phy213752-bib-0018] Motamedi‐Fakhr, S. , R. C. Wilson , and R. Iles . 2017b Tidal breathing patterns derived from structured light plethysmography in COPD patients compared with healthy subjects. Med. Devices 10:1–9. 10.2147/MDER.S119868 PMC5214700 28096696

[phy213752-bib-0009] Newth, C. J. L. , and J. Hammer . 2005 Measurements of thoraco‐abdominal asynchrony and work of breathing in children Pp. 14–145 *in* Hammer J. and Eber E., ed. Paediatric pulmonary function testing. Karger Basel, NY.

[phy213752-bib-0019] Papiris, S. , A. Kotanidou , K. Malagari , C. Roussos . 2002 Clinical review: severe asthma. Crit. Care 6:30–44. 11940264 10.1186/cc1451 PMC137395

[phy213752-bib-0020] Schmalisch, G. , S. Wilitzki , and R. R. Wauer . 2005 Differences in tidal breathing between infants with chronic lung diseases and healthy controls. BMC Pediatr. 5:36. 16150146 10.1186/1471-2431-5-36 PMC1215490

[phy213752-bib-0021] Schmidt, M. , B. Foitzik , R. R. Wauer , F. Winkler , G. Schmalisch 1998 Comparative investigations of algorithms for the detection of breaths in newborns with disturbed respiratory signals. Comput. Biomed. Res. 31:413–425. 9843627 10.1006/cbmr.1998.1493

[phy213752-bib-0022] Sivan, Y. , T. W. Deakers , and C. J. Newth . 1990 Thoracoabdominal asynchrony in acute upper airway obstruction in small children. Am. Rev. Respir. Dis. 142:540–544. 2389905 10.1164/ajrccm/142.3.540

[phy213752-bib-0023] Stick, S. 1996 Measurements during tidal breathing Pp. 117–138 *in* Stocks J., ed. Infant respiratory function testing. Wiley‐Liss, New York, NY.

[phy213752-bib-0024] Stick, S. M. , E. Ellis , P. N. LeSouef , P. D. Sly . 1992 Validation of respiratory inductance plethysmography (“Respitrace”) for the measurement of tidal breathing parameters in newborns. Pediatr. Pulmonol. 14:187–191. 1480445 10.1002/ppul.1950140308

[phy213752-bib-0025] Tauber, E. , T. Fazekas , I. Eichler , C. Eichstill , C. Gartner , D. Y. Koller , et al. 2003 Negative expiratory pressure: a new tool for evaluating lung function in children? Pediatr. Pulmonol. 35:162–168. 12567383 10.1002/ppul.10233

[phy213752-bib-0026] Tobin, M. J. , M. J. Mador , S. M. Guenther , A. Bellocq , C. Straus , and T. Similowski . 1988 Variability of resting respiratory drive and timing in healthy subjects. J. Appl. Physiol. 65:309–317. 3403474 10.1152/jappl.1988.65.1.309

[phy213752-bib-0027] Totapally, B. R. , C. Demirci , B. Nolan , G. Zureikat . 1996 Variability of tidal breathing flow‐volume loops in infants with bronchiolitis. 2332. Pediatr. Res. 39:391.

[phy213752-bib-0028] Weissman, C. , J. Askanazi , J. Milic‐Emili , J. Kinney . 1984 Effect of respiratory apparatus on respiration. J. Appl. Physiol. Respir. Environ. Exerc. Physiol. 57:475–480. 6469818 10.1152/jappl.1984.57.2.475

[phy213752-bib-0029] van den Wijngaart, L. S. , J. Roukema , and P. J. Merkus . 2015 Respiratory disease and respiratory physiology: putting lung function into perspective: paediatric asthma. Respirology 20:379–388. 25645369 10.1111/resp.12480

